# Developmental Transcriptome Analysis of Red-Spotted Apollo Butterfly, *Parnassius bremeri*

**DOI:** 10.3390/ijms231911533

**Published:** 2022-09-29

**Authors:** Kang-Woon Lee, Michael Immanuel Jesse Denison, Karpagam Veerappan, Sridhar Srinivasan, Bohyeon Park, Sathishkumar Natarajan, Hoyong Chung, Junhyung Park

**Affiliations:** 1 Holoce Ecosystem Conservation Research Institute, Hweongsung-gun 25257, Korea; 2 3BIGS Co., Ltd., B-831, Geumgang Penterium IX Tower, Hwaseong 18469, Korea; 3 3BIGS Omicscore Pvt., Ltd., 909 Lavelle Building, Richmond Circle, Bangalore 560025, India

**Keywords:** butterfly transcriptome, *Parnassius bremeri*, endangered, developmental stages, tissue-specific

## Abstract

*Parnassius bremeri* (*P. bremeri*), a member of the genus Snow Apollo in the swallowtail family (Papilionidae), is a high alpine butterfly that lives in Russia, Korea, and China. It is an endangered wildlife (Class I) in South Korea and is a globally endangered species. The lack of transcriptomic and genomic resources of *P. bremeri* significantly hinders the study of its population genetics and conservation. The detailed information of the developmental stage-specific gene expression patterns of *P. bremeri* is of great demand for its conservation. However, the molecular mechanism underlying the metamorphic development of *P. bremeri* is still unknown. In the present study, the differentially expressed genes (DEGs) across the metamorphic developmental stages were compared using high-throughput transcriptome sequencing. We identified a total of 72,161 DEGs from eight comparisons. GO enrichment analysis showed that a range of DEGs were responsible for cuticle development and the melanin biosynthetic pathway during larval development. Pathway analysis suggested that the signaling pathways, such as the Wnt signaling pathway, hedgehog signaling pathway and Notch signaling pathway, are regulated during the developmental stages of *P. bremeri*. Furthermore, sensory receptors were also activated, especially during the larval to adult transition stage. Collectively, the results of this study provide a preliminary foundation and understanding of the molecular mechanism in their transcriptomes for further research on the metamorphic development of *P. bremeri*.

## 1. Introduction

*P. bremeri,* a high-altitude butterfly, is found in Russia, Korea and China. It belongs to the snow Apollo genus (*Parnassius*) of the swallowtail family (*Papilionidae*) but none of the *Parnassius* species have tails. The insect is found in flat, open landscapes, on slopes with forests up to the alpine zone (1500 m) and in forest steppes. It flies in May and June. Some variations in *P. bremeri* were observed due to the geographic isolation and subspeciation through DNA studies, while other variations were related to habitat and effect of temperature on the pupa. The human footprint on our planet is currently threatening biodiversity in all habitats. Probably the greatest threat to biodiversity on our planet is habitat destruction [1]. *P. bremeri* is an endangered butterfly whose population is declining every year due to habitat fragmentation and over-exploitation, as well as a low natural regeneration rate. The disparity in the population of these *Parnassius* butterflies between rural and urban habitats is remarkable [2]. There are almost 58 different species under the genus *Parnassius*, of which *P. bremeri* is critically endangered. Therefore, such an important and endangered species must be protected at all costs. The Apollo butterfly, which includes ten different species, prefers to live in mountain meadows and pastures, especially in cold winters and warm summers. These butterflies also serve as models for the study of metapopulations, population genetics and gene flow [3].

Most previous studies on *P. bremeri* focused mainly on the organization of its mitogenome [4], population studies [5], ecological characteristics, sex pheromones [6] and polyol profiles [7]. Few reports illustrated the importance of population genetics and conservation biology through development of microsatellite markers. Another study, which was based on *P. bremeri* transcriptomes, reported the importance of glycerol accumulation in *P. bremeri* and its role in yielding cold tolerance to the insect [7]. However, still more research is needed on the genomic and transcriptomic aspects for a thorough understanding of the population genetics and species conservation.

Recently, RNA-seq technology has been used as an effective molecular tool to study the evolution of species, determine differentially expressed genes, and examine the population dynamics of organisms over time [8]. With the help of RNA-seq technology numerous studies on arthropods have been done, especially in the order Lepidoptera [9]. Developmental studies have been done on several butterfly species, including *Vanessa cardui* [10], *Pieris rapae* [11], *Bicyclus anynana* [12], and many others. The genome of the nearest species of *P. bremeri, P. apollo, Papilio xutus, Papilio machaon* and *P. bianor* have been studied recently [13,14,15]. In addition, in recent times we have identified antimicrobial peptide candidates in *P. bremeri* against the causative agent of periodontitis, *Porphyromonas gingivalis* [16]. However, the mechanisms involved in the development of *P. bremeri* from egg stage to adult stage remain unclear.

In this study, performed high-throughput transcriptome profiling of eight developmental stages of *P. bremeri*, including the egg (PB), first to fifth instar larva (L1–L5, respectively), adult male (AM) and adult female (AF), and of six adult tissues, namely, antennae (F), head (H), leg (L), wing (W), reproductive organs (R), and body (B). A high-quality transcriptome assembly and annotated transcripts were provided to allow a comprehensive comparison between the eight stages and further elucidate their differences at the gene level. Transcripts related to genes involved in cuticle expression, phenol oxidase expression, juvenile hormone signaling, ecdysone hormone signaling, Wnt signaling, hedgehog signaling, notch signaling, and sensory development were identified and their expressions across developmental stages were investigated. Our transcriptome data expand knowledge of the molecular components of the developmental aspects of *P. bremeri*. As far as we know, this is the first study to analyze transcriptome data from the different developmental stages of *P. bremeri*.

## 2. Results

### 2.1. Sequencing and De Novo Assembly of the P. bremeri Transcriptome

**Figure 1:**
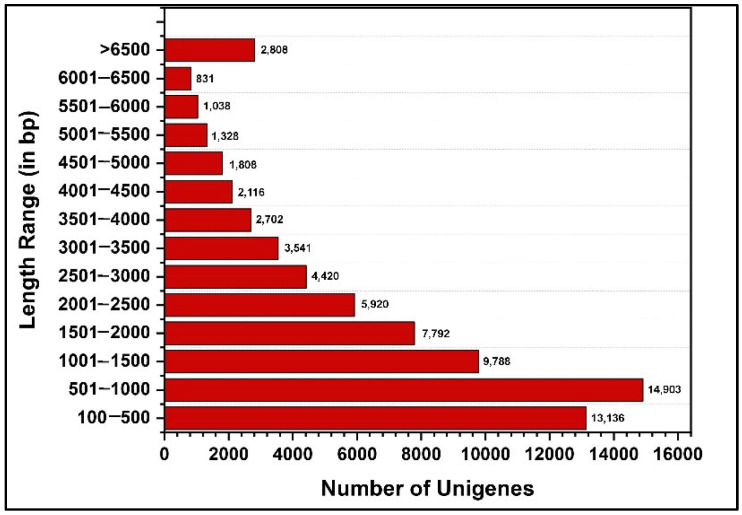
Bar graph showing the differential length distribution of assembled unigenes from the transcriptome. This figure was plotted with number of unigenes as *x* axis against the length of each unigene as *y* axis.

Sequencing of *P. bremeri* tissue yielded 855,693,483 paired 150 bp reads with an average of 25,930,106 reads per sample (Table S1). Raw reads were cleaned by a quality trim (Phred = 0.01) and adaptors were removed. The trimmed high-quality reads were subjected to de novo assembly using Trinity Release v2.14.0, which yielded 72,161 unigenes with an average length of 2063 bp. Of the 72,161 unigenes, 43,663 (60.50%) were greater than 1 kb and 26,542 (36.8%) were greater than 2000 bp (Figure 1).

A BUSCO (Benchmarking Universal Single-copy Orthologs) analysis was performed to determine the completeness of the assembly. BUSCO v5.3.1 revealed a high rate of 965 (95.26%) similar orthologous genes from the Arthropoda ortholog set with few fragmented or missing BUSCOs (Complete gene representation: 94.17% [Single copy: 36%, Duplicates: 58.1%], Fragmented: 1.1%, Missing: 4.8%), indicating a high-quality transcriptome. A summary of the transcriptome composition and quality assessment by BUSCO can be found in Table 1.

### 2.2. Functional Annotation

Among the 72,161 assembled unigene sequences, 46.4% of unigenes showed a similarity distribution of 80–100%, 41.8% of unigenes had 60–80% similarity, and 11.7% of unigenes had 40–60% sequence similarity (≤1 × 10^−5^) to known proteins in the NCBI public databases (nr) (Figure S1A). The distribution of E-values also showed that most unigenes were between 1 × 10^−60^ and 1 × 10^−100^ (Figure S1B). Of the functional annotation of the 72,161 unigenes, 63,782 unigenes yielded BLAST hits, 53,093 had InterProScan IDs (24.4%), 51,255 unigenes with GO mapping (23.5%), 41,314 unigenes with GO annotation (19%), while 8379 of unigenes yielded no BLAST hits (3.8%) (Figure S1C).

Our results showed that the matching efficiency (i.e., sequences with hits) increased with the length of the unigene. Of the unigenes with significant blast hits, 13,063 (~21%) matched the Asian swallowtail butterfly, *Papilio xuthus*, followed by the Old-World swallowtail, Papilio machaon (~15.8%), and the common Mormon, *Papilio polytes* (Figure S1D). Blast results were used for functional categorization of the assembled unigenes. For this step, the Blast2GO annotations were merged with the InterProScan annotations to maximize the number of sequences with assigned functions. A total of 79,867 GO terms were identified for 41,064 annotated sequences among the top three ontologies. Of these sequences, 31,940 (40%) GO terms were assigned to molecular functions (GO:0003674), followed by 26,703 (33.4%) to biological processes (GO:0008150) and 21,224 (26.6%) to cellular components (GO:0005575) (Figure S2).

Within the category of molecular functions, binding (GO:0005488) and catalytic activities (GO:0003824) were the most represented GO terms with 62.2% (19881 sequences) and 56.9% (18186 sequences), respectively. For biological processes, the top GO terms were cellular (GO:0009987, 76%) and metabolic (GO:008152, 66%) processes. In the cellular component category, the predominant GO terms were grouped into membrane (GO:0016020, 65%), membrane part (GO:0044425, 49.7%), cell (GO:0005623, 49%), and cell part (GO:0044464, 49%). GO Enrichment analysis provided information on the regulated GO names in each comparison. Interproscan was used to identify conserved domains or functional units within protein query sequences. A total of 53,093 unigenes (73.6%) were annotated in Interproscan and generated 3557 domains. A KAAS search assigned KEGG orthological terms to 14,374 genes, which were assigned to 419 pathways. A total of 952 genes were assigned to the ‘Metabolic Pathways’ category (KO01100) of KEGG pathways. The numbers of genes assigned to Wnt pathways, Hedgehog pathways, and Notch pathways were 60, 23 and 24, respectively.

### 2.3. Identification of Differently Expressed Genes (DEGs)

**Figure 2:**
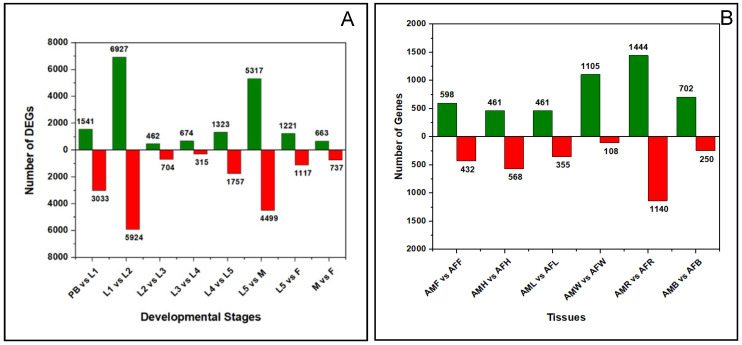
Bar graph of up- and down-regulated genes from pairwise comparisons. This figure shows the number of DEGs that are upregulated (green) and downregulated (red) across the (**A**) developmental stages of *P. bremeri* and (**B**) across the tissues of *P. bremeri*.

Among the 72,161 unigenes, differential expression of genes was analyzed in the biologically relevant comparisons, namely PB vs. L1, L1 vs. L2, L2 vs. L3, L3 vs. L4, L4 vs. L5, L5 vs. AM, L5 vs. AF, and AM vs. AF. The differentially expressed genes in the different libraries were plotted in a volcano plot. The volcano plot shows significance on the *y*-axis and fold change on the x-axis. Fold change (log2FC ≥ 1) and an adjusted *p*-value (≤0.05) were used as thresholds for significance testing (Figures S3 and S4). The statistics of significant genes were plotted as a bar plot in Figure 2.

The number of unigenes differentially expressed in different developmental stages and tissues of both sexes were shown as bar charts (Figure 2, Table S2).

Upset plot analysis (also called Venn) performed for the unigenes in the different developmental stages and tissues showed only 2 common unigenes in the developmental stages and 3 unigenes in the tissue-specific comparison (Figure S5A,B).

The functional enrichment of the GO list of the top 10 unigenes in the comparison of the different developmental stages (Figure S6, Table S3) and tissues of both sexes (Figure S7, Table S3) are shown as a dot plot.

### 2.4. Differentially Expressed Genes (DEGs) across Egg versus First Instar Larval Comparisons

We also compared DEGs across the egg and the first larval instar stages. A total of 4574 genes were differentially regulated, of which 1541 genes (33.69%) were upregulated and 3033 genes (66.31%) were downregulated (Figure 2A, Table S2). The upregulated genes in this comparison showed enrichment in most of the cellular components, like intracellular anatomical structure (GO:0005622), cytoplasm (GO:0005737), intracellular membrane-bounded organelle (GO:0043231) and intracellular organelle (GO:0043229) (Figure S6A, Table S3). In addition, we analyzed the downregulated genes in PB vs. L1 comparison and found that genes which encode for Procathepsin L-like, larval cuticle protein, heatshock 70 kDa protein cognate 4, and facilitated trehalose transporter Tret1 isoform X1 were downregulated. Enrichment of genes responsible for catalytic activity (GO:0003824), oxidoreductase activity (GO:0016491) and transporter activity (GO:0005215) was observed (Figure S6B, Table S3). Facilitated trehalose transporter genes are responsible for the accumulation of trehalose in and out of the cells. We also observed that the members of the glycolysis pathway, such as glucose dehydrogenase, fructose bisphosphate aldolase and glyceraldehyde 3-phosphate dehydrogenase, were downregulated.

### 2.5. Differentially Expressed Genes (DEGs) across Developmental Transition Phases

The comparison groups L1 vs. L2, L5 vs. AM and L5 vs. AF were considered to be the transition phases in the metamorphic development of *P. bremeri*. These three comparisons represent early and late developmental stages in *P. bremeri* development, respectively. We observed that the majority of DEGs were regulated in L1 vs. L2 (6927 unigenes were upregulated and 5924 genes) and L5 vs. AM (5317 upregulated and 4499 downregulated) comparisons (Figure 2).

#### 2.5.1. Initial Transition Phase: First Instar Larval to Second Instar Larval

We found some important unigenes upregulated in the L1 vs. L2 comparison which directly play a major role in the development of *P. bremeri*. Among the unigenes in L1 vs. L2 comparison, those encoding for gastrula zinc finger protein XlCGF57.1-like, gustatory receptor 2 and transposable elements, such as RNA-directed DNA polymerase from mobile element jockey-like, were strongly downregulated.

#### 2.5.2. Final Transition Phase: Fifth Instar Larval to Male Adults and Female Adults

Similarly, we compared the DEGs regulated across the fifth instar larval stages and the adults of both the sexes to understand the induction of adult-specific genes in the fifth instar larval stage. A total of 9816 DEGs (5317 upregulated and 4499 downregulated) and 2338 DEGs (1221 upregulated and 1117 downregulated) were regulated in L5 vs. AM and L5 vs. AF comparisons, respectively. A higher number of DEGs were regulated in the L5 vs. AM than in any other comparison groups (Figure 2). In the L5 vs. AM comparison group, we observed that DEGs encoding the arrestin homolog, which is responsible for the regulation and trafficking of different G-protein-coupled receptors (GPCRs) [17], B-sensitive opsin, chitin synthase 2, ecdysteroid hormone, facilitated trehalose transporter Tret1-like, odorant binding protein, odorant receptors, glutamate receptor ionotropic, leucine-rich neuronal protein, long wavelength-sensitive opsin 2, protein artemis, regulating synaptic membrane exocytosis protein 2 isoform X1, seminal fluid protein HACP004, protein artichoke and several uncharacterized proteins, were upregulated. The downregulated genes encoded for arylphorin subunit alpha-like, endocuticle structural glycoprotein, SgAbd-2-like, facilitated trehalose transporter, members of juvenile hormone signaling pathway, larval cuticle protein, prostaglandin reductase-1 like and xanthine dehydrogenases. Further, Venn analysis showed that 954 genes were commonly upregulated in L5 vs. adult male comparison while 822 genes were commonly downregulated in L5 vs. adult female comparison. The genes encoding for B-sensitive opsin, chitin binding domain protein cbd-1-like isoform, long wavelength-sensitive opsin, membrane alanyl aminopeptidase, arrestin homolog, facilitated trehalose transporter, synaptic vesicle glycoprotein 2C-like and troponin C-isoform 1, were upregulated in both of the comparison groups. Similarly, genes encoding for arylprotein subunit beta-like, hexamerin, larval cuticle protein and superoxide dismutase were downregulated in common.

Most of the development centered genes were harbored in L1 vs. L2 and L5 vs. AM stage comparisons denoting that they were the transition phases during first instar larval and fifth instar larval stages, respectively.

### 2.6. Differential Expressed Genes across the Mid-Larval Developmental Stages (L2 to L5)

We further analyzed the genes differentially expressed in the L2 vs. L3 stage comparison. Among the 1166 DEGs, 462 genes were upregulated, and 704 genes were downregulated (Figure 2A, Table S2). Those genes that encoded protein NDNF-like and cytochrome P450 4C1-like were upregulated, whereas not much deviation in the expression was observed in the downregulated genes (log2FC is ~−2 to −3). The GO enrichment analysis of regulated genes in L2 vs. L3 comparison showed oxidoreductase activity (GO:0016491), tyrosinase activity (GO:0004503), serine-type endopeptidase activity (GO:0004252) and melanin biosynthetic process from tyrosine (GO:0006583). The GO terms enriched by the downregulated genes corresponded to cellular components, like the intrinsic component of membrane (GO:0031224), integral component of membrane (GO:0016021), membrane (GO:0016020) and some transmembrane transporter activity (GO:0055085). In L3 vs. L4, we observed the upregulation of genes that encode cuticle protein, EIA binding protein p400-like, ecdysone induced protein 74EF isoform X1 and odorant receptors. The responsible GO terms enriched from the upregulated genes were structural constituent of ribosome (GO:0042302), integral component of membrane (GO:0016021) and chitin binding (GO:0008061) (Figure S6A, Table S3). Downregulation of pancreatic triacylglycerol lipase in the L3 vs. L4 comparison denoted the catabolic activity of lipases on the triglycerides. However, their complete mechanism is not yet clear. In the L4 vs. L5 comparison, a total of 3080 genes were differentially regulated, of which 1323 genes were upregulated and 1757 genes were downregulated (Figure 2A, Table S2). Some of the genes that regulate Ommochrome-binding protein-like, Superoxide dismutase, acidic and basic juvenile hormone-suppressible protein 2-like and many uncharacterized proteins were upregulated. Superoxide dismutase (SOD) is an important enzyme in insects, especially in the larval stage, for the removal of ROS, which increases the tolerance of *P. bremeri* to oxidative stress, as in the case of *Chironomous riparius* Mg. larvae. Surprisingly, we observed that the expression of SOD was increased during the fourth instar larvae in agreement with the instances of *Chironomous* larva [18]. Among the downregulated genes, we found some of the genes that are responsible for cellular anatomical activity (GO:0110165), internal component of membrane (GO:0016021), structural constituent of cuticle (GO:0042302) and membrane (GO:0016020) (Figure S6B, Table S3).

### 2.7. DEGs in Adult Male (AM) vs. Adult Female (AF)

We studied the DEGs expressed in the adult male vs. adult female comparison in which a total of 1400 genes were differentially expressed. Among 1400 genes, 663 DEGs were upregulated and 737 DEGs were downregulated (Figure 2A, Table S2). We noticed the upregulation of seminal fluid protein, seminal metalloproteinase-1-like and sperm-associated antigen-b-like isoform X2 related to male reproductive function. We observed downregulation of genes which encode cuticle protein, both larval and pupal. We performed GO enrichment analysis on the AM vs. AF comparisons and the top 10 most significant GO terms from upregulated genes are shown in Figure S6A, Table S3, and downregulated genes in Figure S6B, Table S3.

### 2.8. Differential Expression Genes in the Butterfly Tissues of Both the Sexes

We compared the tissues in different groups to find out the sex-specific genes regulated between male and female butterflies. The comparison groups are as follows: adult male feelers (AMF) vs. adult female feelers (AFF), adult male heads (AMH) vs. adult female heads (AFH), adult male legs (AML) vs. adult female legs (AFL), adult male wings (AMW) vs. adult female wings (AFW), adult male reproductive organs (AMR) vs. adult female reproductive organs (AFR) and adult male body (AMB) vs. adult female body (AFB) (Figure 2B, Table S2). We observed a higher expression of genes in the comparison groups, AMR vs. AFR (*n* = 2584) and AMW and AFW (*n* = 1213). In AMR vs. AFR comparison, some of the notable upregulated genes expressed protein takeout-like, protein Wnt-6, odorant receptor 92a, radial spoke head protein 9 homolog, seminal fluid protein HACP044, seminal metalloproteinase, synaptotagmin-5-like, protein Borderless, protein Charlaton and protein gooseberry isoform XI. We found the expression of takeout protein responsible for male courtship behavior and responsible for sex-specific functions [19]. In the downregulated genes, those expressing general odorant binding protein 83a-like, glutamate receptor, larval cuticle protein, zonadhesin-like isoform X1 and X2. GO enrichment analysis was done to represent the top 10 most significant GO terms from upregulated genes (Figure S7A, Table S3) and downregulated genes (Figure S7B, Table S3) in the tissues of both sexes.

### 2.9. Differential Regulation of Larval Cuticle and Its Related Protein

**Figure 3:**
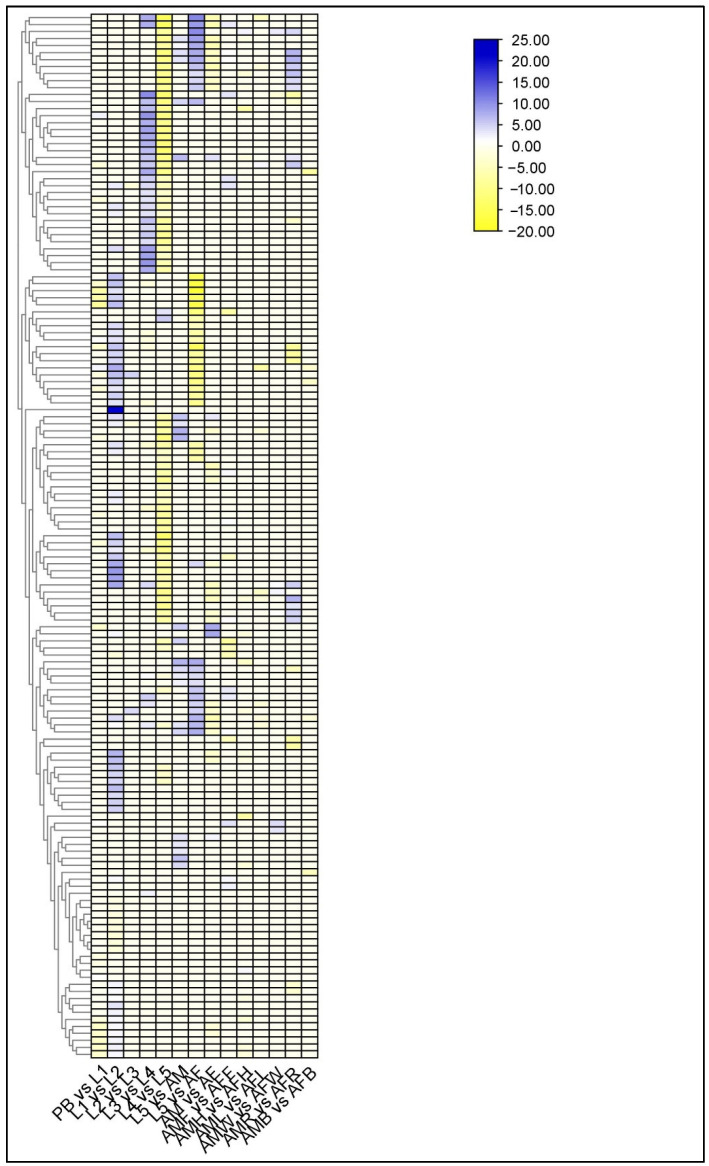
Identification of genes assigned to the cuticle protein in the *P. bremeri* transcriptome. Heatmap representation of the differentially expressed genes of cuticle proteins.

We observed the higher expression of cuticle proteins at the larval stage using heatmap analysis (Figure 3, Table S4). Most of the cuticle proteins were upregulated in L1 vs. L2 comparison group, L3 vs. L4 comparison, L5 vs. AM and L5 vs. AF comparison groups. Interestingly, we found that the expression of cuticular proteins were alternatively switched on and off by different sets of genes between larval stages (L1 to L5). We also examined the expressed genes in adult tissues and found cuticle proteins being expressed in AMR vs. AFR, AMW vs. AFW and AMF vs. AFF. We noticed that the cuticle was highly expressed in the male reproductive system, more so than in the females. In AMW vs. AFW and AMF vs. AFF comparison groups, only a few cuticular proteins were expressed.

**Figure 4:**
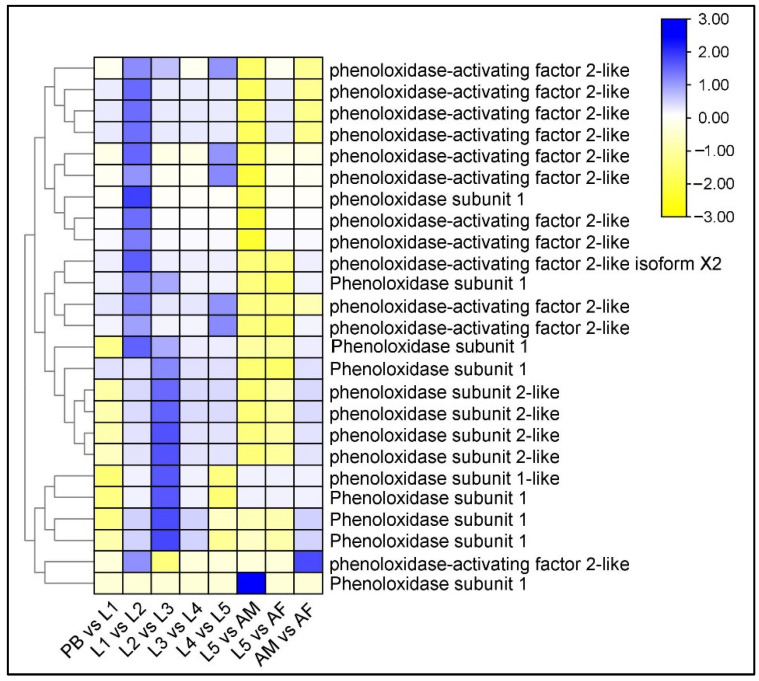
Identification of DEGs of Phenol oxidases in *P. bremeri* transcriptome. Heatmap representation of the differentially expressed genes of phenol oxidases.

We observed the upregulated expression of phenol oxide subunit-1 and phenol oxidase activating factor-2 like protein in the comparison groups L1 vs. L2 and L2 vs. L3, i.e., the early larval stages of *P. bremeri* (Figure 4, Table S5). The enrichment of GO terms, melanin biosynthetic process from tyrosine (GO:0006583) and melanin biosynthetic process (GO:0042438) defined 8 sets of genes that encode phenoloxidase subunit 1 in the L2 vs. L3 comparison groups.

### 2.10. Differential Regulation of Juvenile-Hormone Signaling Genes

**Figure 5:**
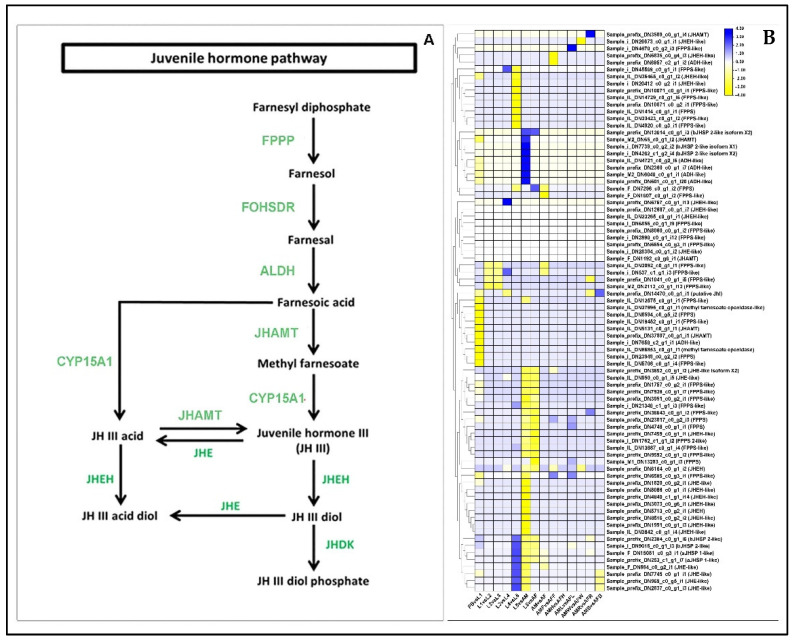
Identification of genes assigned to the Juvenile Hormone biosynthetic (JHB) pathway in the *P. bremeri* transcriptome. KAAS was used to identify genes annotated by BLAST against the specific insect database and assigned to a KEGG pathway (**A**) JH biosynthetic pathway components/gene. Colored boxes indicate the presence of the genes identified. A green colored box signifies the presence of an annotated gene that does not show a change in expression levels. (**B**) Heat map showing the log2-fold change of the FPKM values of the DEGs.

The genes encoding farnesyl diphosphate synthase (FPPS), farnesol dehydrogenase (FDH), juvenile hormone acid methyltransferase (JHAMT), methyl farnesoate epoxidase (CYP15A1), Juvenile hormone epoxide hydrolase-like (JHEH), and Juvenile hormone esterase-like (JHE1) coding were manually curated and assigned to the Juvenile hormone metabolic pathway at each stage and tissue comparison [20]. In the overall comparisons, a total of 118 DEGs were downregulated, whereas 71 DEGs were upregulated with respect to the members of the JH signaling pathway. In the PB vs. L1 stage comparison, the majority of DEGs (25 unigenes) were downregulated, whereas only 2 DEGs were upregulated (Table S6). All the members of the juvenile hormone signaling pathway were significantly downregulated in the PB vs. L1 comparisons. In L1 stage, the DEGs for FPPS, JHE and JHEH were significantly upregulated compared to L2. In L2 vs. L3 comparison, we noted that the genes encoding FPPS were downregulated (Figure 5).

### 2.11. Differential Regulation of Ecdysone Receptor (EcR) Family Genes

**Figure 6:**
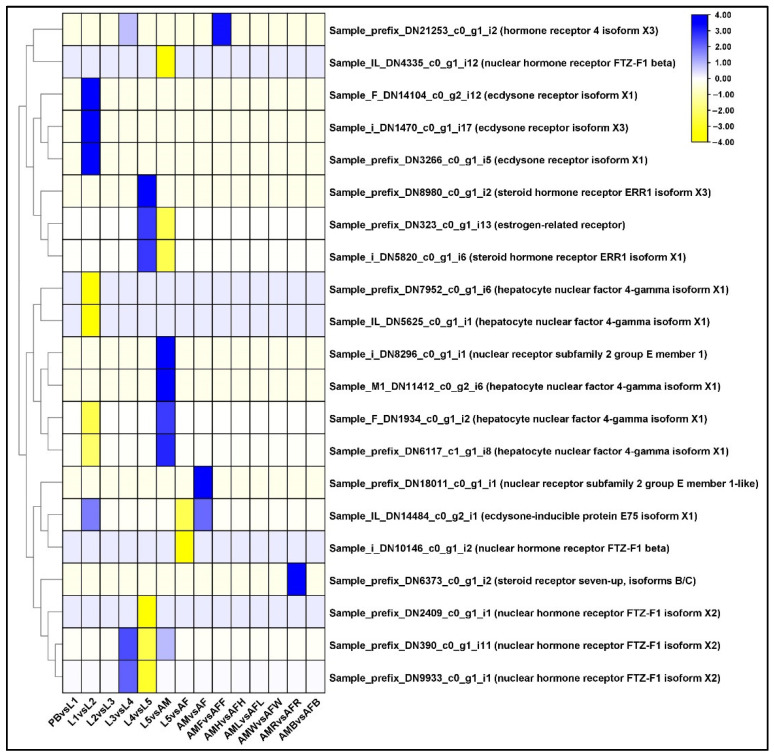
Identification of genes assigned to the Ecdysone biosynthetic (EH) pathway in the *P. bremeri* transcriptome. KAAS was used to identify genes annotated by BLAST against the specific insect database and assigned to a KEGG pathway. Heat map showing the log2-fold change of the FPKM values of the DEGs.

We found that a total of 18 and 16 DEGs of the ecdysteroid family were upregulated and downregulated, respectively. In L1 vs. L2 comparison, 3 unigenes encoding ecdysone receptor were upregulated and those encoding hepatocyte nuclear factor were downregulated. In L4 vs. L5 comparisons, the DEGs encoding the ecdysone hormone receptor were low (Figure 6). In AM vs. AF stage, the expression of ecdysone receptor was upregulated (Table S7). The presence of two probable nuclear hormone receptor HR3 isoform X1s was induced by the 20-hydroxyecdysone (20E) as a delayed early response.

### 2.12. Differential Regulation of Sensory Receptors

**Figure 7:**
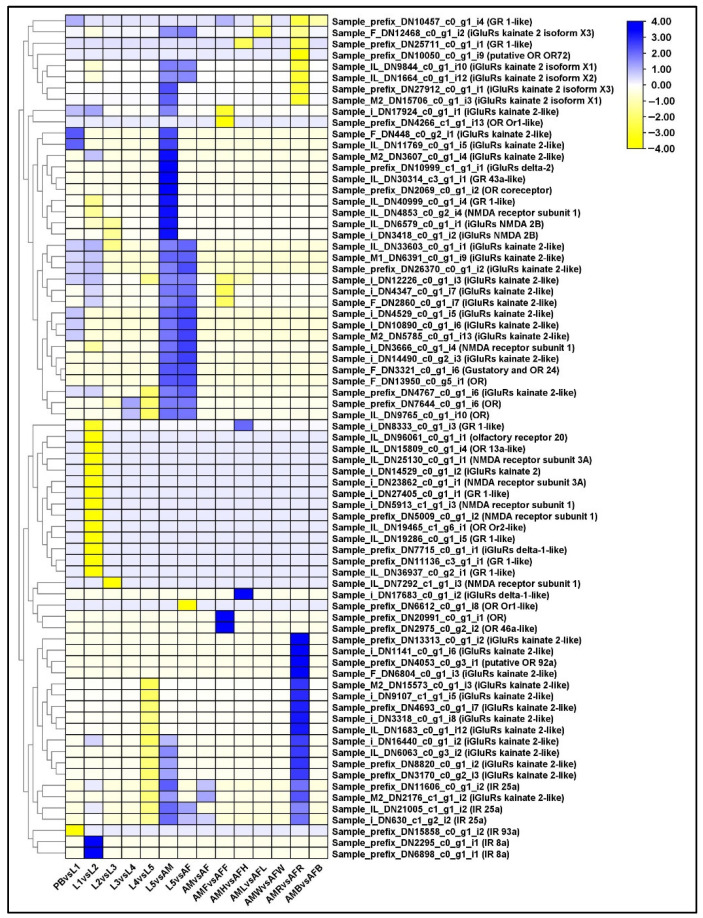
Identification of genes assigned to the Sensory receptors in the *P. bremeri* transcriptome. Heatmap representation of the differentially expressed genes of Sensory receptors.

In PB vs. L1 comparison group, some the ionotropic glutamate receptor, kainate 2-like (Grik2) were upregulated. In L1 vs. L2 comparison, we found upregulation of only a few IR8as. IR25a and IR8a are co-receptors which are highly conserved among Lepidoptera. In the comparison group L5 vs. AM, we noticed major expression of Grik2 and IR8. This suggested that Grik2 plays an important role in the late developmental phase of *P. bremeri*. In Drosophila, it was observed that, compared to larval stages, adults showed distinct transcriptional profiles of olfactory system [21]. In adults the ORs were clearly expressed in antennae (or) feelers. Therefore, we analyzed across the tissues of *P. bremeri* of both the sexes and found differential expression of ORs in their feelers. In AMF vs. AFF comparison, DEGs responsible for the expression of modifier of MDG4 were upregulated. The downregulated unigenes encode for putative protein phosphatase 2C 71, unnamed protein product and uncharacterized protein. However, the function of putative phosphatase 2C 71 is still unknown. Most of the upregulated DEGs in AMR vs. AFR comparison, encode for Grik2 which is responsible for neuronal development (Table S8). A heatmap representation of DEGs related to the expression of sensory receptors is shown in Figure 7.

### 2.13. Differential Regulation of Pathways in Wing and Eye-Spot Development

#### 2.13.1. Wnt-Signaling Pathway

**Figure 8:**
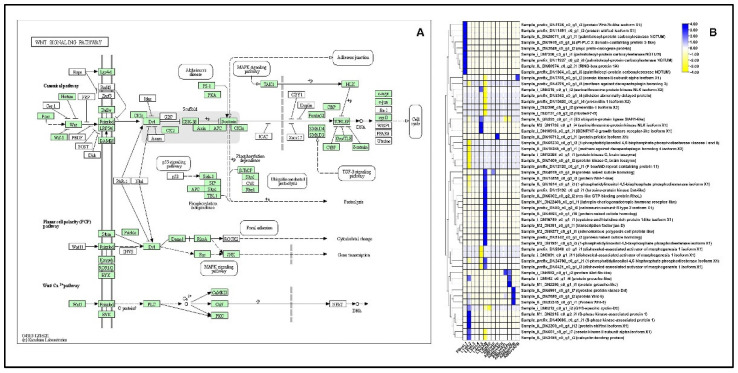
Identification of genes assigned to the Wnt signaling pathway in the *P. bremeri* transcriptome. KAAS was used to identify genes annotated by BLAST against the specific insect database and assigned to a KEGG pathway (**A**) Wnt signaling pathway components/genes. Colored boxes indicate the presence of the genes identified. A green colored box signifies the presence of an annotated gene that does not show a change in expression levels. (**B**) Heat map showing the log2-fold change of the FPKM values of the DEGs.

Overall statistics show that a total of 43 DEGs were upregulated and 41 DEGs were downregulated in the developmental stages and tissues of both sexes that corresponded to Wnt-signaling in *P. bremeri*. In the PB vs. L1 comparison, 9 DEGs were upregulated, encoding Wnt-7b protein, Shifted Isoform X1 protein, RING-box protein, *myc* protooncogene, and palmitoleoyl protein carboxylesterase NOTUM. In the L1 vs. L2 comparison, 11 DE genes were upregulated, and 12 genes were downregulated. Down-regulated DEGs included those encoding Wnt-1-like protein kinase, palmitoleoyl protein carboxylesterase NOTUM, and F-box protein, whereas Wnt-7b protein, S-phase protein kinase, and Wnt-6 were up-regulated. When L4 and L5 were compared, a total of 9 DEGs were expressed, of which 4 were upregulated and 5 were downregulated. When comparing L5 and AM, the 15 DEGs encoding tyrosine protein kinase Dnt-like, Wnt-4, protein naked cuticle homolog, and 1-phosphatidylinositol 4,5-bisphosphate phosphodiesterase isoform X1 [*Papilio xuthus*] were upregulated (Figure 8, Table S9).

#### 2.13.2. Hedgehog Signaling Pathway

**Figure 9:**
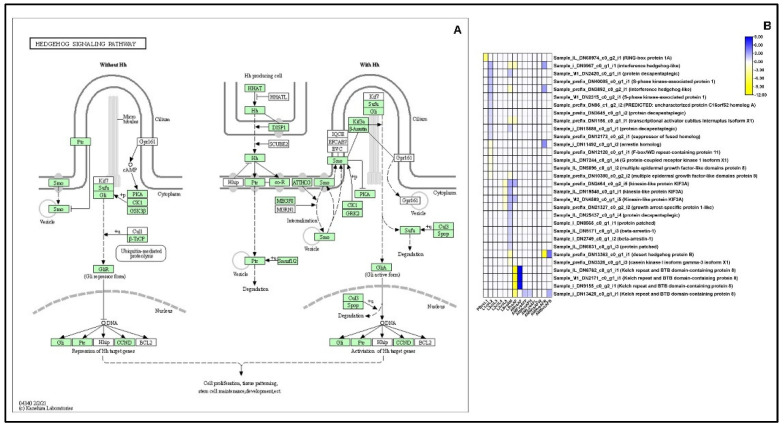
Identification of genes assigned to the Hedgehog signaling pathway in the *P. bremeri* transcriptome. KAAS was used to identify genes annotated by BLAST against the specific insect database and assigned to a KEGG pathway (**A**) Hedgehog signaling pathway components/genes. Colored boxes indicate the presence of the genes identified. A green colored box signifies the presence of an annotated gene that does not show a change in expression levels. (**B**) Heat map showing the log2-fold change of the FPKM values of the DEGs.

A total of 33 DEGs and 23 DEGs were upregulated and downregulated in the Hedgehog signaling pathway, respectively, Particularly, in L1 vs. L2 comparisons, DEGs expressing interference Hedgehog, protein decapentaplegic and S-phase kinase associated protein were upregulated, whereas arrestin homolog, F-box/WD repeat and GPCR kinase 1 isoform X1 were downregulated. Similar expressions were observed in the L5 vs. AM and L5 vs. AF comparisons, suggesting that these DEGs are involved in the regeneration of legs at the late larval or pupal stage (Figure 9, Table S10).

#### 2.13.3. Notch-Signaling Pathway

**Figure 10:**
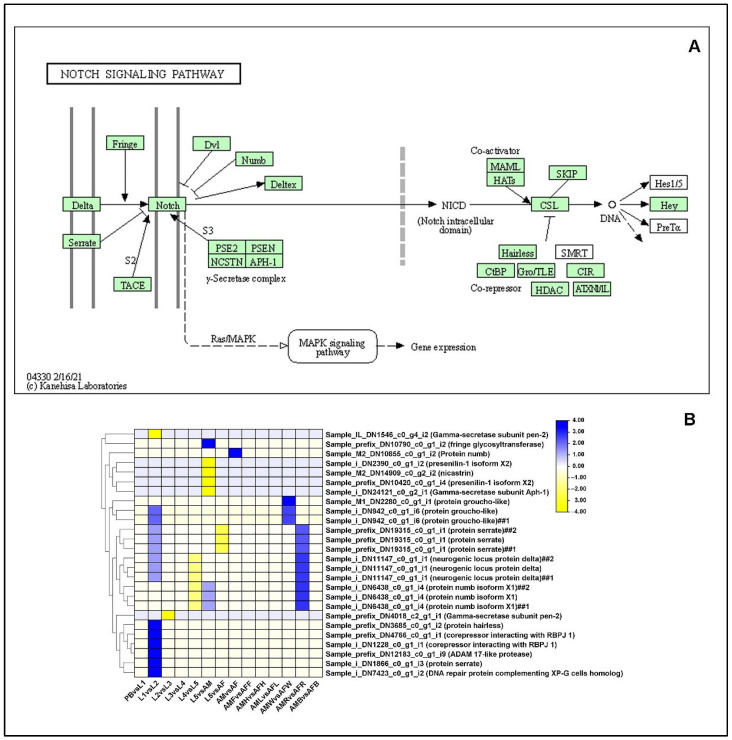
Identification of genes assigned to the Notch signaling pathway in the *P. bremeri* transcriptome. KAAS was used to identify genes annotated by BLAST against the specific insect database and assigned to a KEGG pathway (**A**) Notch signaling pathway components/genes. Colored boxes indicate the presence of the genes identified. A green colored box signifies the presence of an annotated gene that does not show a change in expression levels. (**B**) Heat map showing the log2-fold change of the FPKM values of the DEGs.

We examined the differential expression of the genes enriched in the Notch signaling pathway. In the L1 vs. L2 comparison, 10 unigenes were differentially regulated, 9 of which were upregulated and 1 was downregulated. Mainly, protein serrate was upregulated in L1 compared to L2 stage. One of the important roles of the Notch signaling pathway is the development of wing primordium, especially wing blade and margin. In the L5 vs. AM comparison, a total of 6 unigenes were differentially regulated, 2 DEGs being upregulated and 4 DEGs being downregulated. Protein Presenilin was downregulated in L5 compared to the AM stages. Presenilin is an important factor which stabilizes the C-terminal fragment of the amyloid precursor protein in higher animals, whereas in Drosophila, it is proteolytically cleaved and broadly expressed with the highest levels of neurons within the larval CNS. It was also shown that mutations in presenilin led to defects in eye and wing development. In the AMW vs. AFW comparison, 2 unigenes were upregulated, whereas in the AMR vs. AFR comparison, 3 unigenes were upregulated. In butterfly larvae, the notch signaling pathway has been known to regulate the formation of pigment, especially camouflage formation in larvae. We could observe that the titers of ligand Notch remained constant throughout the developmental stages, whereas another ligand, Serrate, was expressed in the mid, (L3), and late, L4 and L5, larval stages (Figure 10, Table S11).

## 3. Discussion

*P. bremeri*, the red-spotted butterfly, is an endangered species worldwide, and research on its genome or transcriptome could be beneficial for the development of conservation strategies. Few researchers have focused on developing molecular markers, such as SSRs [22]. Our previous transcriptome study dealt with the identification of antimicrobial peptide candidates against the primary periodontitis pathogen *Porphyromonas gingivalis* [16]. In general, the genus *Parnassius* is an altitudinal butterfly, and they are thought to appear in different colors depending on altitude. There are more than 58 species under this genus, belonging to the family *Papilionideae*. To date, limited genome information is available on this species, although it is on the Korean Red List of Threatened Species and is regionally protected by law as an endangered species [22]. Recently, in 2021, the complete genome assembly of *Parnassius apollo* was completed in Germany [13]. *P. bremeri*, the red-spotted Apollo butterfly, like other butterflies and moths, goes through four developmental stages to complete its life cycle: egg, larva, pupa, and adult. The egg is the most primitive stage in the butterfly’s development, and any influence of environmental factors on the egg ultimately affect the growth of subsequent developmental stages. Although diapause is prevalent during the pupal and adult stages in 110 lepidopterans, diapause in the egg is common in Apollo butterflies of the genus *Parnassius* [15]. Eggs laid early during the winter season are likely to overwinter [23]. During this egg stage, most genes probably control basic metabolic pathways for cellular development. The second stage, the first larval stage, is the transition between the nonfeeding egg stage and the feeding larval stage. Larval stages consist of five instances from L1 to L5 [7], as shown in Figure 1. During the larval stages, they have a higher feeding rate and increase their body size from small to large, as well as developing a high reproductive capacity. *P. bremeri* exhibit day feeding behavior during the larval stage to promote their development [7]. It has also been suggested that growth is slow in the larval stage and accelerates after reaching an ambient temperature. The larva of the Apollo butterfly feeds on the perennial plant *Sedum kamtschaticum*. Normally, in butterflies and moths (*Lepidoptera*), environmental factors, such as weight gain and tissue coloration, play an important role in larval stage development [24]. After reaching the fifth larval stage, the genus *Parnassius* develops into a moth-like pupa surrounded by a silken cocoon [25]. The butterfly also develops its wings in the pupa and uses the hook to escape from the loosely wrapped cocoon. These hooks are present only in *Parnassius* butterflies, while they are absent in other genera. The hook is useful only in the soft vein stage, while it is useless in the fully hardened adults. The final developmental stage of *P. bremeri* is the imago, where the adults hatch from the pupa, and their fluttering is less in proportion to the speed with which they move. Adult Parnassius butterflies are known to move slowly. Adult males usually patrol in search of adult females. Males are known to attach a structure called a sphragis to females to prevent females from mating again. Larvae and adults are reportedly venomous to predators [26]. To understand the overall behavior, it is necessary to examine the transcriptomic architect of *P. bremeri*.

In this study, the complete transcriptome data of the developmental stages and the tissues of both sexes of *P. bremeri* was obtained. The significant genes were identified using the *p*-value and the fold change thresholds in the eight comparisons of developmental stages and eight comparisons of tissues of both sexes. GO enrichment analysis was performed to understand the DEGs relevant to the biological process, molecular function and cellular components. KEGG pathway analysis also showed the regulation of significant pathways corresponding to the developmental stages and our findings are discussed. Several transposable elements (TEs), such as piggyBac transposable element derived protein 2-like, retrovirus-related Pol polyprotein from transposon TNT 1–94, reverse transcriptase, RNA-directed DNA polymerase from mobile element jockey, transposon Tf2-9 polyprotein, Ty3-G and Ty3-I were encoded during the embryonic stage. These TEs occupy a major portion of the insect genome and account for the huge variety in insect genome sizes [27]. In such a way, TEs are vital during embryonic development [28], in phenotypic plasticity [29], and diapause [30]. Even though regulation of TEs was witnessed throughout the insect development [31], higher transcriptional activity of TEs was observed at the egg stage, just like *Locusta migratoria* [29]. TEs are believed to exist to inactivate genomic regions important for early embryonic regulation as an alternative mechanism to DNA methylation [32]. However, intense research is needed to elucidate the importance of TEs during embryonic stage. We observed that the cuticle was expressed at all developmental stages of *P. bremeri*. However, the same DEGs of the cuticle were not expressed, but alternative circuits of different sets of genes were expressed. The cuticle of successive larval stages is generally quite similar, but differs from that of the adult stage, which is characterized by sexual maturity and the development of functional wings [33]. This shows that cuticle characteristics differ during the metamorphic evolution of the butterflies. We believe that the differential expression of several sets of genes at different stages is due to the molting behavior of holometabolous insects, in which the old cuticle is shed and a new cuticle is formed [33]. Such molting behavior in insect larvae is controlled by the steroid hormone ecdysteroids, which we discuss later on [34]. In adults, cuticular protein is regulated in the testes of most Lepidoptera insects, such as *Spodoptera litura* [35]. DEGs also regulate wing development through expression of histidine-rich cuticular protein [36] in adults.

The larvae appear dark colored because they have a pigmented cuticle. Cuticle development and pigmentation depend on several factors, including the involvement of phenoloxidases in the melanization pathway, and regulation by juvenile hormones and ecdysone hormones [37]. For instance, aldo-keto reductase AKR2E4, which is responsible for the reduction of 3-dehydroecdysone to ecdysone in the silkworm *Bombyx mori* L. [38], developmentally regulated arylphorin subunit [39], chorion peroxidase involved in the chorion cross-linking [40], members of juvenile hormone signaling pathway [41] and ecdysone signaling pathway [42], and most of them belong to the cuticle development. Phenoloxidases, are initially formed as inactive zymogens (prophenoloxidases), which are later converted into active phenoloxidases [43]. Phenoloxidases play an important role in the process of melanogenesis, converting phenols to quinones, and these quinones are, subsequently, polymerized to melanin [44]. Therefore, we found that the *P. bremeri* larva utilizes the melanization pathway described by Soderhall and Cerenius [45]. The reaction steps include the following: (1) hydroxylation of phenylalanine to tyrosine by phenylalanine hydroxylase, (2) hydroxylation of tyrosine by phenol oxidase to form DOPA, (3) oxidation of DOPA to dopaquinone and 5,6-dihydroxyindole (DHI) to indole-5,6-quinone. These indole quinones are polymerized to eumelanins and, in the presence of thiols, to pheomelanins [43]. Further research will be done in future to prove this concept. The melanization pathway is usually controlled by the jasmonic acid signaling cascade and ecdysone regulating signaling cascade. FPPS is involved in the synthesis of farnesyl diphosphate by catalyzing the head to tail condensation of two molecules of isopentenyl diphosphate with dimethylallyl diphosphate to form farnesyl pyrophosphate (FPP) [46]. In butterflies of the Papilionideae family, the first to fourth instar larva appear as mimics of bird droppings, whereas at the fifth instar larval stage they attain a camouflaged form among the leaves of the host plant. Such a developmental switch is believed to be controlled by the juvenile hormone pathway [47]. The concentration of juvenile hormone was inversely proportional to the development of larval stage to adult stage, i.e., at the fifth larval stage there was a decrease in the juvenile hormone level to aid in the development of morphological structure. The juvenile-hormone related genes were studied for differential regulation. Of the enzymes that are involved in JH degradation, JHEH hydrolyzes JH to JH acid. JHEH causes irreversible opening of the Juvenile hormone epoxide ring converting it into JH diol [48,49]. Both these enzymes thereby decrease the titer of JH in the cells promoting larva to adult metamorphosis in *P. bremeri* (Figure 5, Table S6).

In multicellular organisms, most of the developmental transitions are driven by steroid hormones (ecdysteroids). These hormones, in conjunction with the juvenile hormone (JH), regulate development, metamorphosis, reproduction, and aging [50]. Prothoracic glands secrete a kind of steroid, ‘ecdysone’ and get transported to the target organs via hemolymph. The lipophilic nature of ecdysone means it pools directly into the cytoplasm of the cells at the site of development [51,52]. In insects, ecdysteroids play a major role in CNS right from the larval stage to adult stage and are mostly detected in larval neurons [53]. Ecdysteroids, in conjunction with juvenile hormone, cause butterflies to display seasonal polyphenism, which is one of their major aspects [54,55]. We examined the expression of EcR in the developmental stages and tissues of both sexes of *P. bremeri*. The genes regulating the ecdysone pathway were collected from the KEGG pathway. It has been reported that 20E is responsible for pigmentation and expression of melanin biosynthetic genes, especially the yellow color formation ion in the larva of Papilio xuthus [47]. According to earlier studies, 20E induced HR3 receptor is required for developmental progression through early metamorphosis stages, L1 and L2 [56]. The ecdysteroid 20E has also been reported to trigger the ecdysis in butterfly larva. In *Drosophila melanogaster*, ftz-f1 mutants displayed embryonic lethal phenotypes in the first and second larval ecdysis and before pupation (Figure 6, Table S7).

Most insects, including butterflies, use plants for shelter and food, by which they contribute to the development of the terrestrial ecosystem [57]. They possess chemoreceptors to detect the chemical signals that are important for numerous insect ecological behaviors, such as mating, foraging, and oviposition [58]. Among the sensory receptors, olfactory receptors play an important role in the recognition of chemical signals, supporting host validation and acceptance [59]. Olfactory receptors are the highly efficient and precise recognition system that belongs to the olfactory system [60]. In Drosophila, it was observed that, compared to larval stages, adults showed distinct transcriptional profiles of the olfactory system [21]. Some sensory receptor related genes that encode for general odorant binding protein and glutamate and ionotropic receptors were also upregulated, especially protein slit, a conserved odorant receptor protein to detect flowering plant cues [57]. In adults the ORs were clearly expressed in antennae (or) feelers. Therefore, we analyzed across the tissues of *P. bremeri* of both the sexes and found differential expression of ORs in their feelers. In insects, ionotropic receptors have been known to be evolved from ionotropic glutamate receptors (iGluRs), a conserved family of synaptic ligand-gated ion channels [61]. The major role of ionotropic receptors in insects is the detection of phagostimulants, pheromones [62], salt [63], and volatiles [64] and are believed to be involved in hearing [65]. Therefore, there are different types of chemosensory receptors, gustatory receptors (GRs), olfactory receptors (ORs) and ionotropic receptors (IRs). In the AMF vs. AFF comparison, DEGs responsible for the expression of modifier of MDG4 were upregulated. The downregulated unigenes encode for putative protein phosphatase 2C 71, unnamed protein product and uncharacterized protein. However, the function of putative phosphatase 2C 71 is still unknown. Most of the upregulated DEGs in the AMR vs. AFR comparison encode for Grik2, which is responsible for neuronal development (Table S8). Grik2, or the ionotropic receptor (IR25a), is known to be involved in the binding of the excitatory neurotransmitter L-glutamate, causing a conformational change that leads to the opening of the cation channel that converts the chemical signal into an electrical impulse. Binding of an agonist to the receptor desensitizes and inactivates it [66]. In cotton bollworm *Helicoverpa armigera*, IR25a was shown to be expressed in the accessory glands, like the ejaculatory duct and bursa copulatrix [67].

The most conserved genes in animals, *Wnt* genes, are responsible for the various developmental processes. *Wnt* genes play an important role in wing development, starting with axis formation and ending with the mature wing. *Wnt* genes are also involved in the development and evolution of color patterns on butterfly wings [68]. We examined genes belonging to the Wnt signaling pathway to determine if there are candidate genes expressed in the developmental stages and tissues of both sexes of *P. bremeri*. The protein wntless is responsible for the production of Wnt proteins from signaling cells. It is required for wingless patterning processes in Drosophila [69] and has been found in significant reduction of the Hh or Wg signaling pathway. JH plays an important role in regulating insect growth and metamorphosis. Despite the suggestion that JH is involved in the regulation of 20E, it has also been found to regulate the Wnt signaling mechanism in insects. Expression of Wnt7 was first detected in the thoracic and abdominal segments in the lateral regions. In Drosophila melanogaster and *Tribolium castaneum* (*T. castaneum*). Wnt7 was also observed in the central nervous system of *T. castaneum* [70,71]. In another study, four *Wnt* genes (1,6,7,11) were expressed during wing development in *Zerene cesonia* [68]. Downregulation of Wnt7b indicates its increased expression at the L1 stage, which is crucial for abdominal and thoracic development. Several canonical pathways, including planar cell polarity (PCP) signaling pathways, are controlled by Wnt ligands. The protein Wingless and DWnt4 play a vital role in regulating the PCP pathway. The role of PCP signaling pathway in neuronal migration, neuronal polarization, axonal and dendritic guidance and synaptogenesis is notable [72]. In the Mediterranean fruit fly, *Ceratitis capitata*, protein Wnt5b was expressed in flies with full flight ability and regulated cell proliferation in the wings. Under-expression of Wnt5b resulted in flightlessness [73] (Figure 8, Table S9).

Another important signaling pathway in the development of the red-spotted apollo butterfly is the Hedgehog (Hh) signaling pathway. The Hh-signaling pathway has been known to be responsible for wing and eye-spot development in insects [74]. Mainly interference hedgehog is known to activate the hedgehog signaling pathway in *Drosophila melanogaster* [75]. The expression patterns of hedgehog, wingless (wg), and decapentaplegic (dpp) have been observed during leg regeneration of the cricket *Gryllus bimaculatus* [76]. In an experiment, those mutants lacking hh function reduced its expression and had reduced wing size and eye spot. Therefore, the expression of hh is responsible for wing and eye spot development (Figure 9, Table S10).

The notch signaling pathway plays a major role in various developmental processes, such as tissue patterning, cell proliferation and cell fate differentiation [77]. The transmembrane protein encoded by the notch signaling is highly conserved in the animal kingdom [78]. In a study on the role of Notch and Delta ligands in the formation of camouflage in caterpillars, two ligands were suggested to be involved in camouflage formation. The expression level of the protein Serrate was equal to the expression levels of Delta [57]. The differential expression of Notch isoforms was analyzed in the tissues of male and female adults of *P. bremeri*. The gene encoding Gamma-secretase subunit pen-2 was highly expressed across all the tissues, especially in both male and female feelers. The expression of secretase Pen-2 is required for the normal development of *P. bremeri*. The orthologs of secretase enzyme in humans play a major in maintaining a healthier brain, while the absence of Pen-2 has led to Alzheimer disease pathogenesis [79]. The role of this enzyme in the Notch signaling pathway is poorly understood in butterflies. From these results, the significance of the notch signaling pathway in the development of olfactory neurons and wing development was clear [80] (Figure 10, Table S11). Taken together, our data offers insight into the genes responsible for the formation of cuticle, coloration of cuticle, and alternative switching of cuticle formation between larval and adult stages, and the involvement of juvenile hormone and ecdysteroids to support the melanization reaction. Further, we explored the regulation of Wnt signaling, Hedgehog signaling and notch signaling in the metamorphic development of *P. bremeri*.

## 4. Materials and Methods

### 4.1. Sample Preparation

Rearing of *P. bremeri* was carried out under field conditions, as previously reported [7]. Eggs, larvae, male and female adults of *P. bremeri* were collected from August to November 2018 in Hweongsung, Korea (37°31′33.1″ N 128°09′00.3″ E). No chemical or biological insecticides were used in the area during the two months prior to sampling. In brief, eggs were artificially deposited on fallen oak leaves in a double net cage (0.1 × 0.3 mm mesh) for 180 days. The egg-studded leaves were then transferred to a cage for juvenile larvae (40 × 50 × 70 cm), and the newly hatched larvae were collected in a plastic Petri dish (10 cm diameter × 4 cm height) with an addition of the host plant *Sedum kamtschaticum*. At the 4th instar stage, larvae were divided into 30 individuals each and kept in a metal cage (71 × 51 × 88 cm, covered with a 1 × 1 mm metal grid) with host plants. The following samples were included in the study: Egg (PB), first instar larva (L1), second instar larva (L2), third instar larva (L3), fourth instar larva (L4), fifth instar larva (L5), adult male (AM), adult female (AF) representing developmental stages while adult male feelers (AMF), adult female feelers (AFF), adult male head (AMH), adult female head (AFH), adult male wings (AMW), adult female wings (AFW), adult male reproductive organs (AMR), adult female reproductive organs (AFR), adult male body (AMB) and adult female body (AFB) that represents tissues of both sexes. Whole body samples were frozen in liquid nitrogen and stored in the freezer at −80 °C for subsequent RNA analysis. The developmental stages and the duration required by *P. bremeri* are shown schematically in Figure S8.

### 4.2. RNA Extraction and Sequencing

Total RNA was extracted from each samples using Trizol reagent (Invitrogen, Carlsbad, CA, USA) according to the manufacturer’s instruction. The integrity of the isolated RNAs was analyzed using the Bioanalyzer 2100 system (Agilent technologies, Inc., Santa Clara, CA, USA). The RNA integrity count (RIN) > 7 was set as RNAQC to proceed to the next step of cDNA library construction (Truseq Stranded mRNA Prep Kit (Illumina Technologies, San Diego, CA, USA)). Following, the generated cDNA libraries were sequenced using an Illumina NovaSeq 6000 platform to generate 150 bp paired end reads. The raw reads were submitted to NCBI SRA with submission IDs being listed in Table S1.

### 4.3. Assembly Normalization and Quality Assessment

The raw reads of the 14 samples were filtered to remove poor quality reads and reads with adapter sequences before assembly using Trimmomatic software (version 0.39) with default settings [81]. These high-quality reads were prepared for de novo transcriptome assembly using Trinity software (version 2.14.0) with default parameters [82]. In short, Trinity combined specific lengths of overlapping reads and paired-end information to assemble longer fragments that generated transcripts and unigenes that were subjected to annotation analysis. The contigs were merged according to a similarity criterion of 95% in CD-HIT-EST (version 4.6.3) [83]. The contigs were then translated into coding protein sequences using Transdecoder (version 2.0.1) after the longest ORFs were identified. The completeness of the assembled transcriptome was analyzed using BUSCO (v5) on the gVolante web server [84]. An overview of the complete de novo sequencing and analysis of the *P. bremeri* transcriptome is shown in Figure S9.

### 4.4. Functional Annotation and GO Enrichment

Assemblies were aligned with the NCBI nonredundant protein sequence by searching the BLASTX database in the OmicsBox v2.0.36 program [85], and only the best homologue was reported. Gene Ontology Mapping (GO) was performed against the Gene Ontology database integrated in OmicsBox. Sequences that showed significant similarity to known protein sequences in BLASTX searches (E < 1 × 10^−10^) were identified using the InterProScan 5.0 online tool.

The OmicBox program was used to assign Gene Ontology (GO) terms to the annotated sequences to predict the functions of the unique sequences. Here, the e-value hit filter was set to 1 × 10^−3^, the annotation cutoff was set to 55, and the evidence code was set to 0.8 for the different categories as implemented in OmicsBox. BLASTX output was used to run Interproscan, GO mapping to functionally annotate the unigenes. Gene Ontology plots were generated using WEGO v2.0 [78]. Gene ontology enrichment analysis was performed in the OmicsBox with the DEGs in each comparison with the full dataset by implementing false discovery rate (FDR) correction for multiple testing.

### 4.5. Analysis of Differentially Expressed Genes (DEGs)

The developmental stages of *P. bremeri* were compared in pairs: PB vs. L1, L1 vs. L2, L2 vs. L3, L3 vs. L4, L4 vs. L5, L5 vs. AM and AF and AM vs. AF. And tissue-specific comparisons were AMF vs. AFF, AMH vs. AFH, AML vs. AFL, AMW vs. AFW, AMR vs. AFR and AMB vs. AFB. The up-regulated and down-regulated DEGs were selected if log2FC ≥ 1 and log2FC ≤ −1, respectively, with an adjusted *p*-value ≤ 0.05 [86]. DEGs in common and unambiguously regulated at all stages and tissues were analyzed using the Upset Plot in TBtools [87]. The differentially expressed genes specific to the expression of ABC transporters and sensory receptors were identified based on Pfam domain while the members of hormone signaling pathways (Juvenile, Wnt, Hedgehog and Notch) were identified using KEGG Automatic Annotation Server (KAAS) by the assignment method of ‘bi-directional best hit’ against data of Arthropod dataset in the KEGG database [88].

## 5. Conclusions

Our study on the transcriptomic regulation of *P. bremeri* metamorphosis clearly shows that certain unigenes were regulated across the developmental stages and in specific tissues. The generated resource from this study is helpful in laying foundations to understand the precise nature and development of other *Parnassius* butterflies. This study can be used to improve adaptive management plans geared towards species conservation. Pathway identification and candidate gene exploration are important steps toward a better understanding of the complex mechanisms behind the development of butterflies. Further, validation of the expression of candidate genes could improve the conservation of *P. bremeri*.

## Figures and Tables

**Table 1:** De Novo Transcriptome Assembly statistics of *P. bremeri* transcripts and de novo assembled transcripts completeness assessment results using BUSCO analysis.

Measure	Value
Number of genes	72,161
Number of transcripts	124,158
Average contig length (bp)	1043.2
Median contig length (bp)	627
Total assembled bases	75,278,310
GC content of unigene (%)	45.43
Minimum sequence length (bp)	297
Maximum sequence length (bp)	48,967
Number of genes > 1 kb	44,218
Number of genes > 5 kb	472
N50 (bp)	1560
**Features**	**Results**
Complete BUSCOs (C)	954 (94.17%)
Complete + Partial	965 (95.26%)
Complete and single-copy BUSCOs (S)	365 (36%)
Complete and duplicated BUSCOs (D)	589 (58.1%)
Fragmented BUSCOs (F)	11 (1.1%)
Missing BUSCOs (M)	48 (4.8%)
Total BUSCO groups searched	1013
BUSCO version	BUSCO_v5.3.1
Selected ortholog set	Arthropoda

## Data Availability

Raw data associated with this study are accessible through the NCBI Sequence Read Archive (SRA) database under BioProject IDs PRJNA716449 (https://www.ncbi.nlm.nih.gov/sra/PRJNA716449, accessed on 4 March 2022), PRJNA647953 (https://www.ncbi.nlm.nih.gov/sra/PRJNA647953, accessed on 4 March 2022) and PRJNA716454 (https://www.ncbi.nlm.nih.gov/sra/PRJNA716454, accessed on 4 March 2022).

## Supplementary Materials

The supporting information can be downloaded at: https://www.mdpi.com/article/10.3390/ijms231911533/s1.
