# Supplementary material for: Developmental Transcriptome Analysis of Red-Spotted Apollo Butterfly, Parnassius bremeri
Source: Int J Mol Sci. 2022 Sep 29;23(19):11533. doi: 10.3390/ijms231911533 (PMC9569764; doi:10.3390/ijms231911533)
Supplement: Supplementary file 1 [file ijms-23-11533-s001.zip › ijms-1887952-supplementary.pdf]

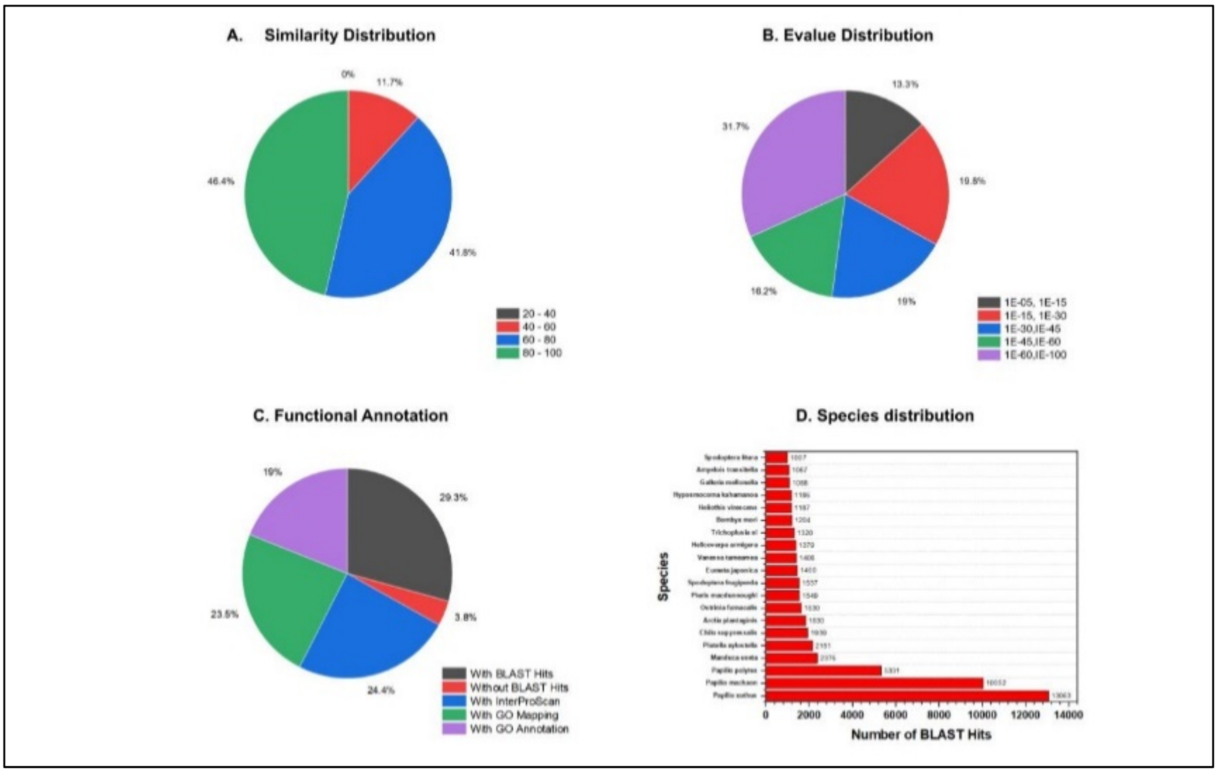

Figure S1. Characteristics of the alignment of *P. bremeri* unigenes against the NR database. A) Similarity distribution of the BLAST hits obtained for each unigene, B) E-value distribution of the BLAST Hits, C) data distribution of annotated and non-annotated sequences, D) Top-hit species distribution for unigenes from the transcriptome of *P. bremeri*.

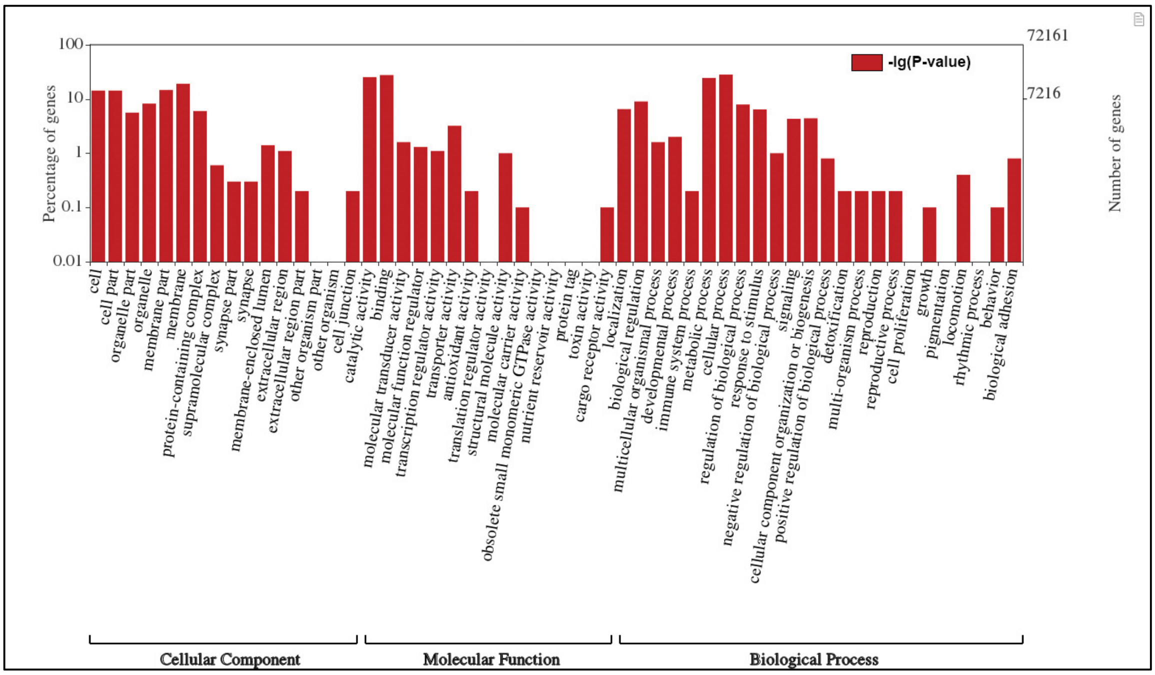

Figure S2. GO categories of *P. bremeri* all-unigenes. This figure illustrates the summary of cellular component, biological process, and molecular function. Under each category (the third GO-level), the percentage (left y-axis) and total number (right y-axis) of all-unigenes are shown.

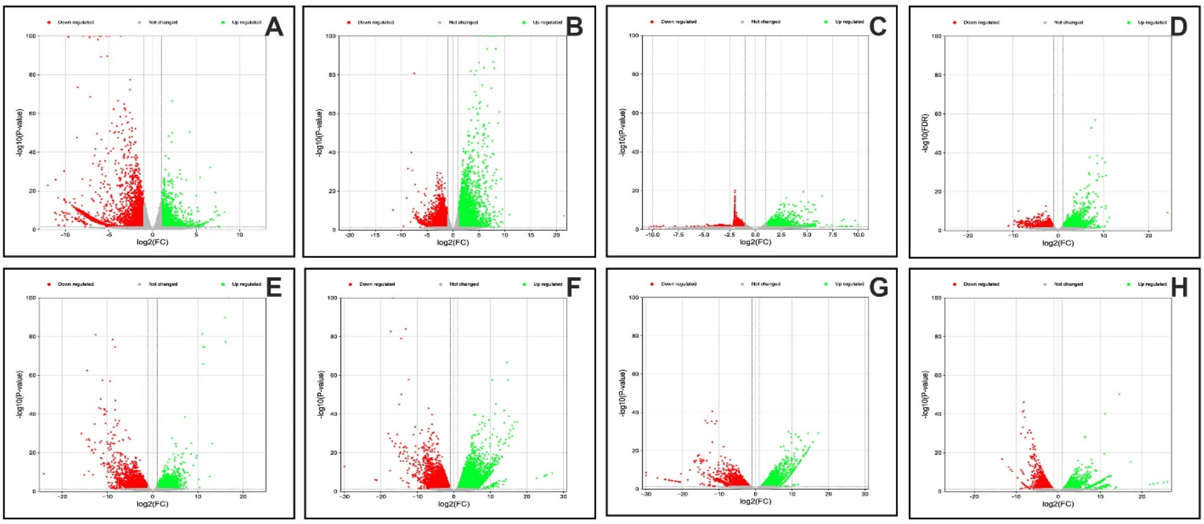

Figure S3. Volcano Plot representation of differentially regulated genes across *P. bremeri* developmental stages. This figure represents the volcano plots showing fold-change and p-value for the comparisons across developmental stages : A) PB vs L1, B) L1 vs L2, C) L2 vs L3, D) L3 vs L4, E) L4 vs L5, F) L5 vs AM, G) L5 vs AF and H) AM vs AF. Upregulated genes are shown in green color and the downregulated genes are shown in red color.

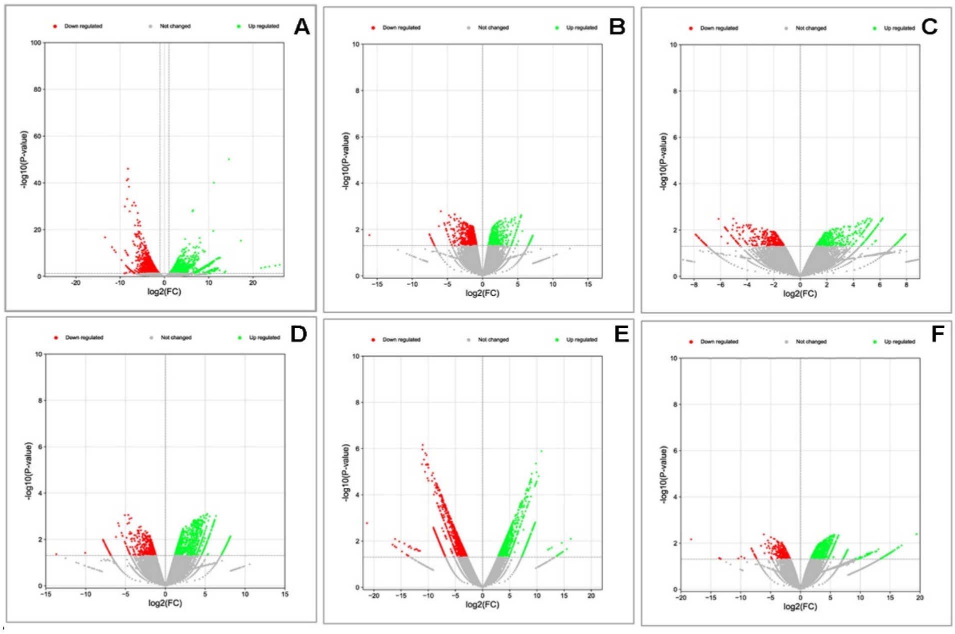

Figure S4. Volcano Plot representation of differentially regulated genes across *P. bremeri* tissues of both sexes. This figure represents the volcano plots showing fold-change and p-value for the comparisons across tissues of both sexes : A) AMF vs AFF, B) AMH vs AFH, C) AML vs AFL, D) AMW vs AFW, E) AMR vs AFR, F) AMB vs AFB. Upregulated genes are shown in green color and the downregulated genes are shown in red color.

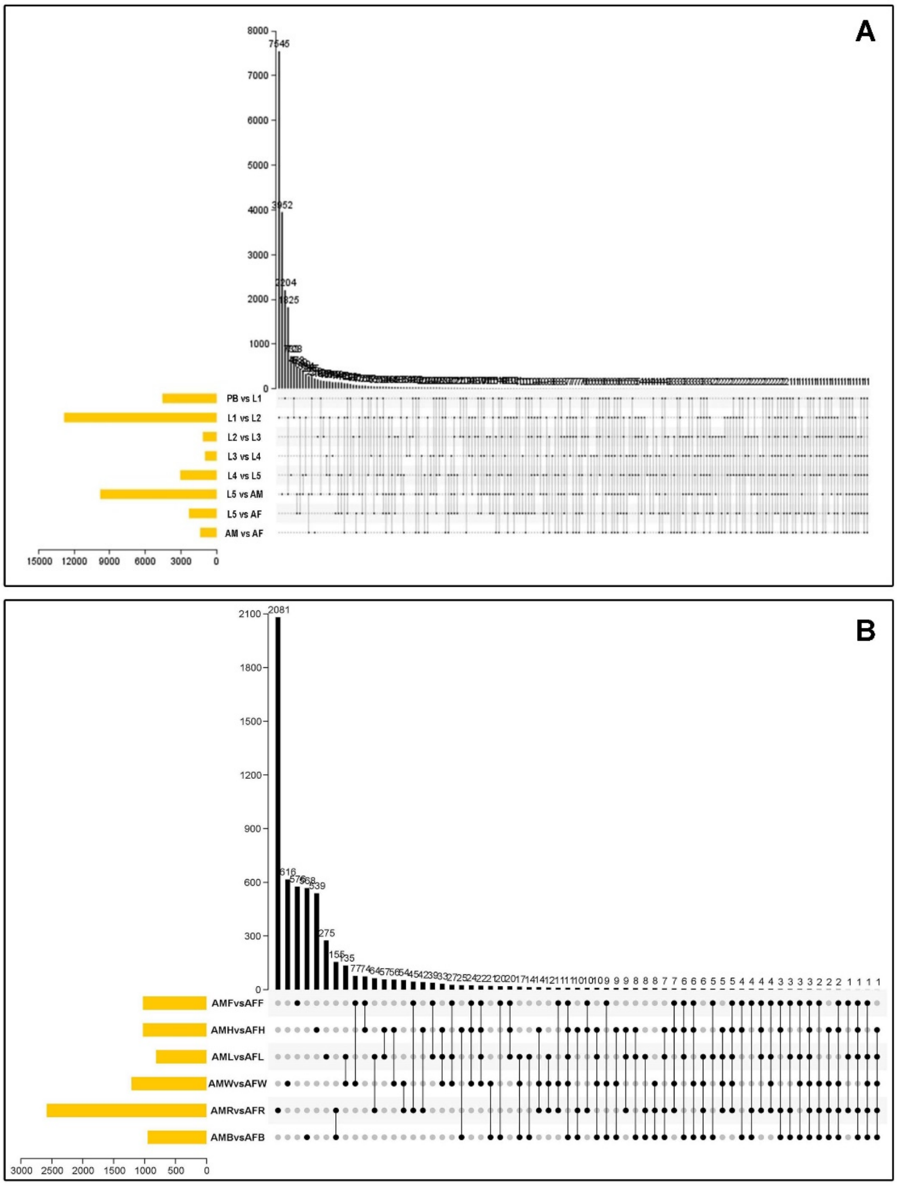

Figure S5. Venn diagram representation of commonly regulated DEGs across different comparisons of developmental stages (A) and tissues of both sexes (B) of *P. bremeri.*

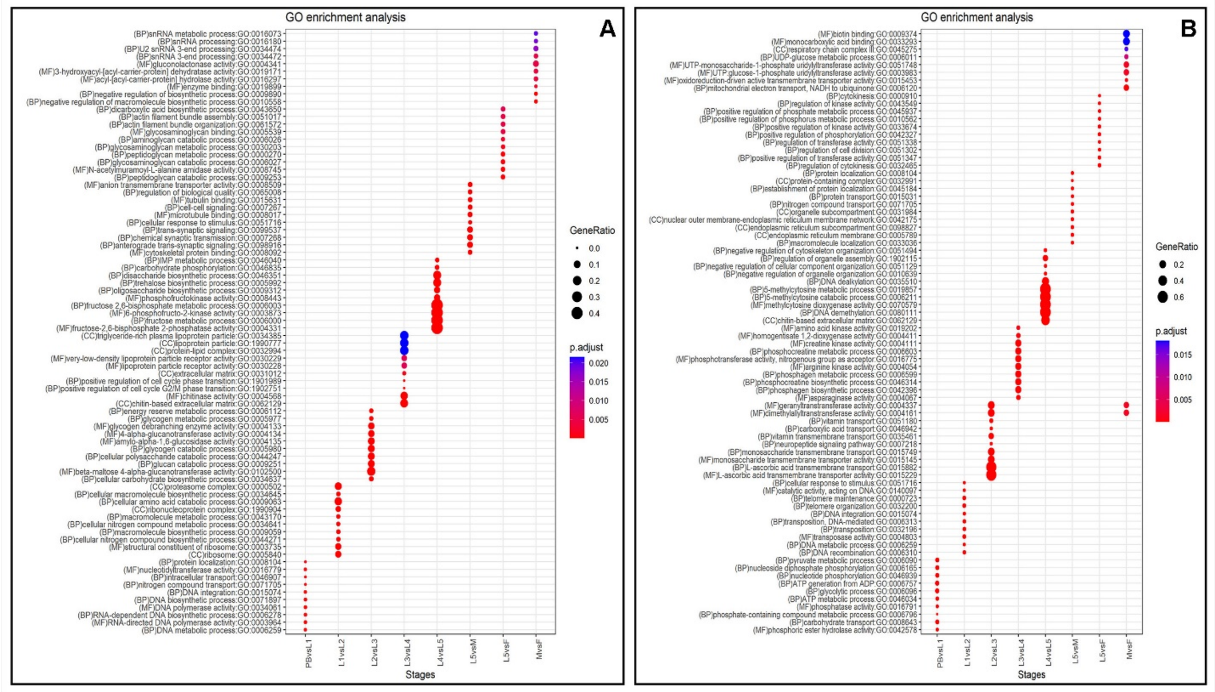

Figure S6. Dot plot of GO term enrichment showing the top 10 enriched GO Terms from A) Upregulated DEGs and B) Downregulated DEGs across development stages of *P. bremeri*. The colors indicate the adjusted p-values (FDR) from Fisher's exact test, and dots size is proportional to the gene ratio of differentially expressed genes (DEG) in the background of reference set in the given pathway.

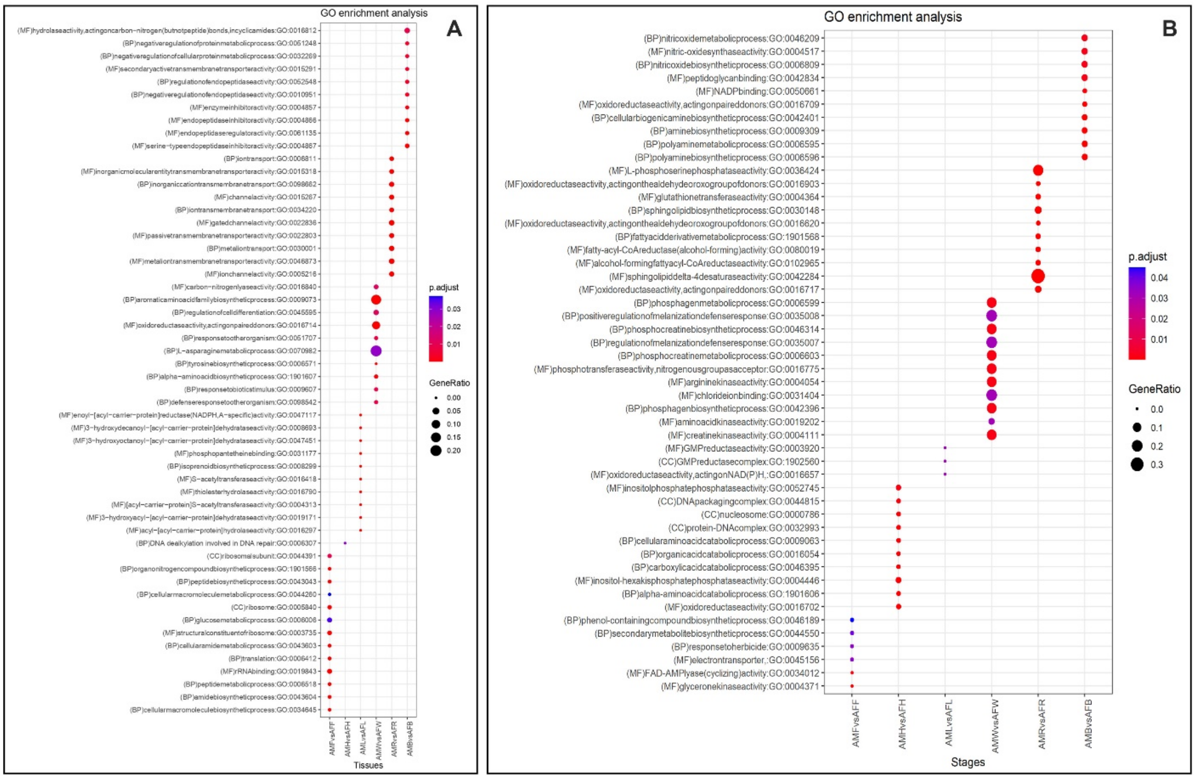

Figure S7. Dot plot of GO term enrichment showing the top 10 enriched GO terms from A) Upregulated DEGs and B) Downregulated DEGs across different tissues of *P. bremeri*. The colors indicate the adjusted p-values (FDR) from Fisher's exact test, and dots size is proportional to the gene ratio of differentially expressed genes (DEG) in the background of reference set in the given pathway.

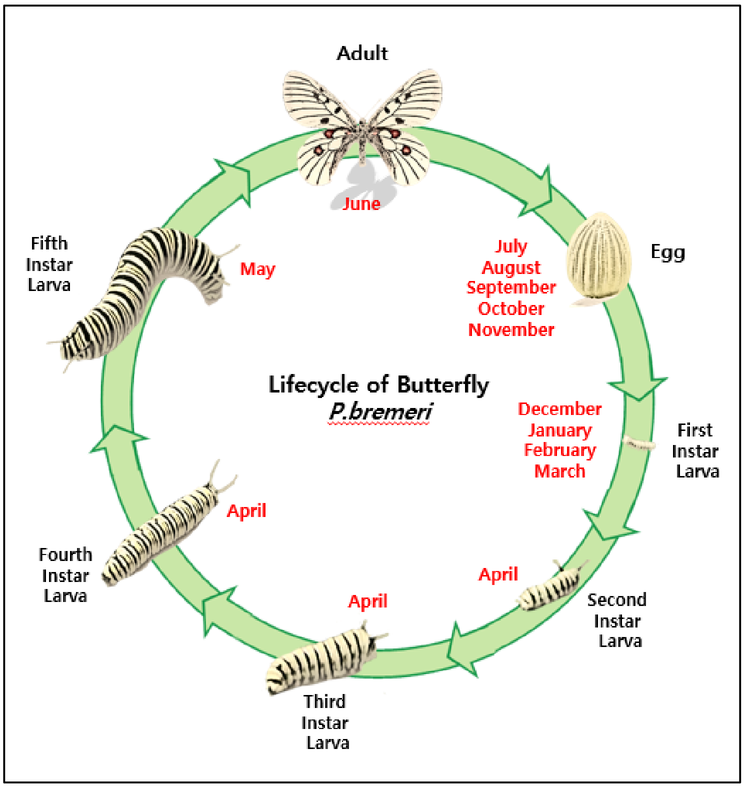

Figure S8. Metamorphic stages of *P.bremeri*. This figure shows the metamorphic development of Red-Spotted Apollo butterfly at 6 different stages, namely Egg (180 days after ovipulation), first (120 days), second (10 days), third (10 days), fourth (10 days), fifth (30 days) instar larval stages and Adult Stages (30 days). The month in red color included with the figure denotes the time-duration taken for metamorphosis development of butterfly, *P.bremeri*.

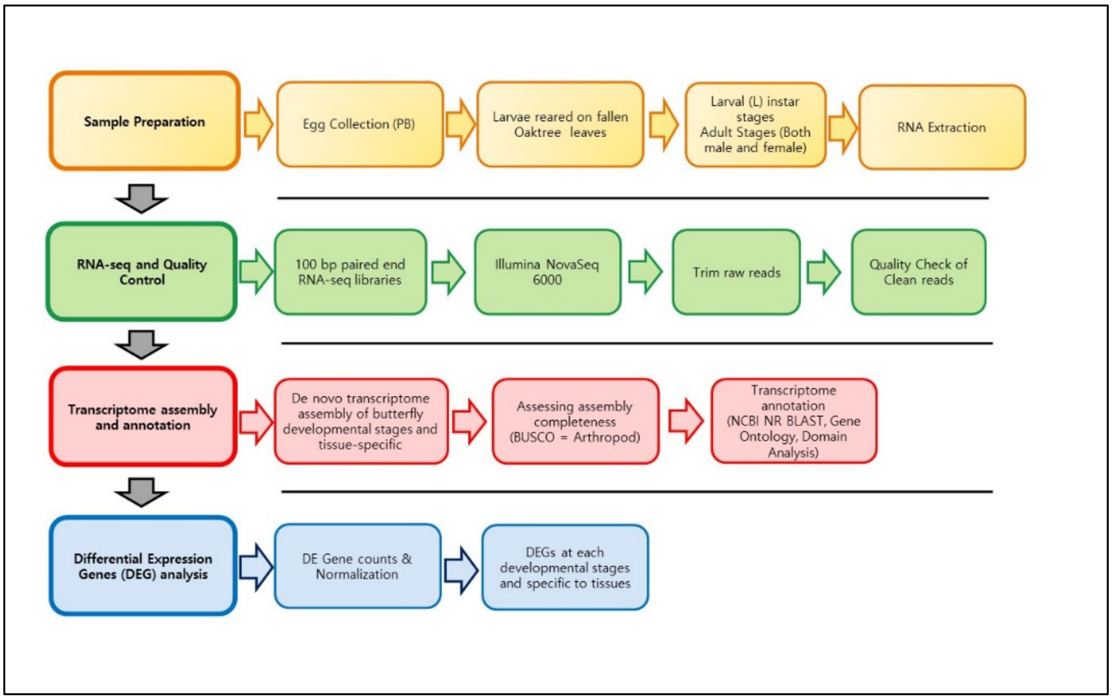

Figure S9. Overview of RNA-seq analysis workflow. This figure represents the workflow adopted for the thorough analysis of transcriptomes of *P. bremeri*. The top to bottom workflow direction denotes the main categories and left to right workflow direction denotes the sub-categories.
